# A novel and simple Inflation-deflation method with inflated targeted segments for confirming the intersegmental border under video-assisted thoracoscopic surgery: a technical note

**DOI:** 10.1186/s13019-026-04606-8

**Published:** 2026-07-24

**Authors:** Wei Chen, Zhanwu Yu

**Affiliations:** Department of Thoracic Surgery, Cancer Hospital of Dalian University of Technology, Liaoning Cancer Hospital & Institute, 44 Xiaoheyan Road, Dadong District, Shenyang, 110042 China

**Keywords:** Inflation-deflation, Segmentectomy, Intersegmental border, Targeted segment artery

## Abstract

With the increase in the diagnosis rate of small pulmonary nodules, the segmentectomy has been increasingly used in clinical practice worldwide. However, for the segmentectomy, the confirmation of intersegmental plane has always puzzled many thoracic surgeons. This paper described a new method, named artery-based Inflation-deflation method, used to confirm the intersegmental border under video-assisted thoracoscopic surgery. After only dividing the targeted segment artery and preserving the targeted segmental veins and bronchi, the intersegmental plane was successfully generated by this novel Inflation-deflation method. Compared with other current methods, this new technique had some advantages including the smaller airway damage, easier to determine the intersegmental plane, the higher fault-tolerant rate and easy to operate without any additional devices. And a further clinical study should be carried out to verify the feasibility and applicability of this new technique.

## Introduction

With the advancement of medical imaging technique and the extensive application of low-dose computed tomography, the detection rate of early-staged non-small cell lung cancer (NSCLC) has risen remarkably in recent decades. Meantime, ground-glass opacity (GGO) and multiple lung nodules account for a considerable proportion. Since 1960, for early-staged NSCLC, surgery is still the mainstay of treatment, and lobectomy remains a standard surgical procedure [1]. However, sub-lobar resection, such as segmentectomy or wedge resection, is increasingly favored by thoracic surgeons now. According to National Comprehensive Cancer Network (NCCN) guidelines, for selected patients with peripheral T1ab, N0 tumors, sublobar resection should be strongly recommended. For those with insufficient pulmonary reserve or other significant comorbidities that contraindicate lobectomy, segmentectomy is preferred and more suitable alternative to wedge resection [2]. Due to the lack of obvious anatomical boundaries of the pulmonary segments, many thoracic surgeons are discouraged from the segmentectomy. Recently various methods were introduced to solve this problem [3–12]. Here, we will assess the clinical feasibility of a novel and simple Inflation-deflation method with resecting the targeted segmental artery and preserving the targeted segmental bronchi by discussing a case.

## Aim

Herein, a novel and simple Inflation-deflation method was introduced by our team to confirm the intersegmental border under uniportal video-assisted thoracoscopic surgery (VATS). Compared with existing methods, this new method is easier to manage, easier to spread and does not need any additional auxiliary materials.

### Case presentation

Representative case.

**Fig. 1:**
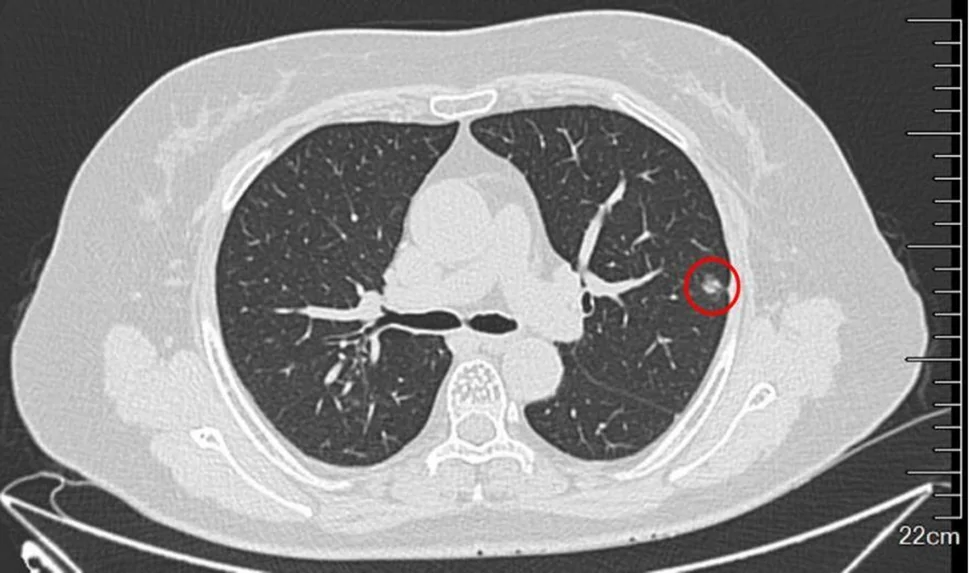
Findings on high-resolution computed tomography. The lung lesion was marked by red circle

**Fig. 2:**
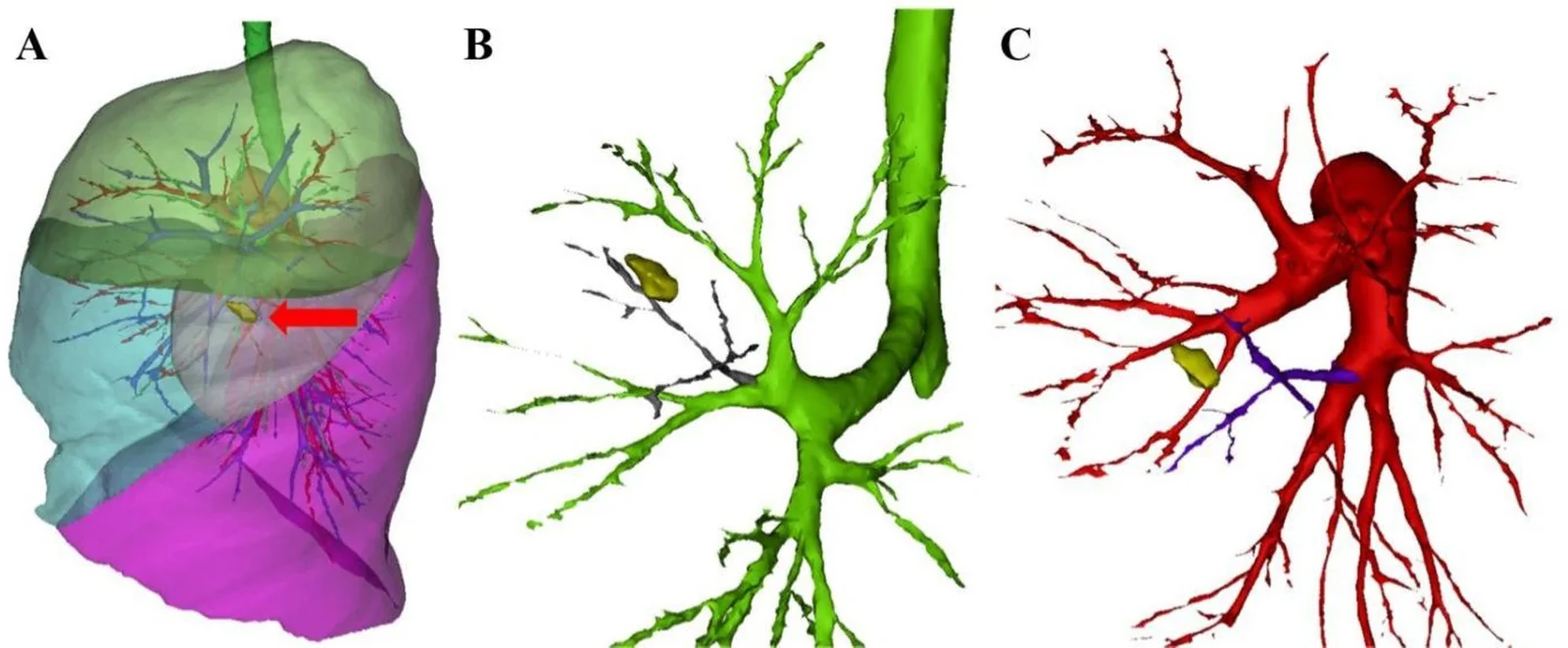
Three-dimensional simulation. (**A**) The lung lesion (marked with a red arrow) was located in superior lingular segment. (**B**) The superior lingular bronchus was marked in grey, the rest were green. (**C**) The superior lingular branch of left pulmonary artery was marked in purple, the rest were red

A 64-year-old woman did examination due to precordial discomfort. The computed tomography scan showed a 6 millimeters solitary pulmonary nodule (SPN) in left upper lobe (LUL) with rough edges and pleural indentation (Fig. 1). The three-dimensional simulation (Fig. 2A) showed this SPN was located in superior lingular segment (Segment 4, S4). As showed in Fig. 2B, the superior lingular bronchus (Bronchus 4, B4) lay between the inferior lingular bronchus and upper division of left upper lobe bronchus. There was anatomical variance in the artery and the superior lingular branch of left pulmonary artery (Artery 4, A4) was originated from the common basal artery (Fig. 2C).

She had a past history of angina pectoris for 20 years and hypertension for 5 years. And she could achieve complete remission of angina pectoris with Suxiao Jiuxin Pills and nitroglycerin in oral way. Evaluation by the Department of Cardiology and Anesthesiology, there is no contraindications to surgery but high cardiovascular risk during the perioperative period. After Multiple Disciplinary Team discussion, our team performed uniportal VATS S4 segmentectomy for her.

## Technique

**Fig. 3:**
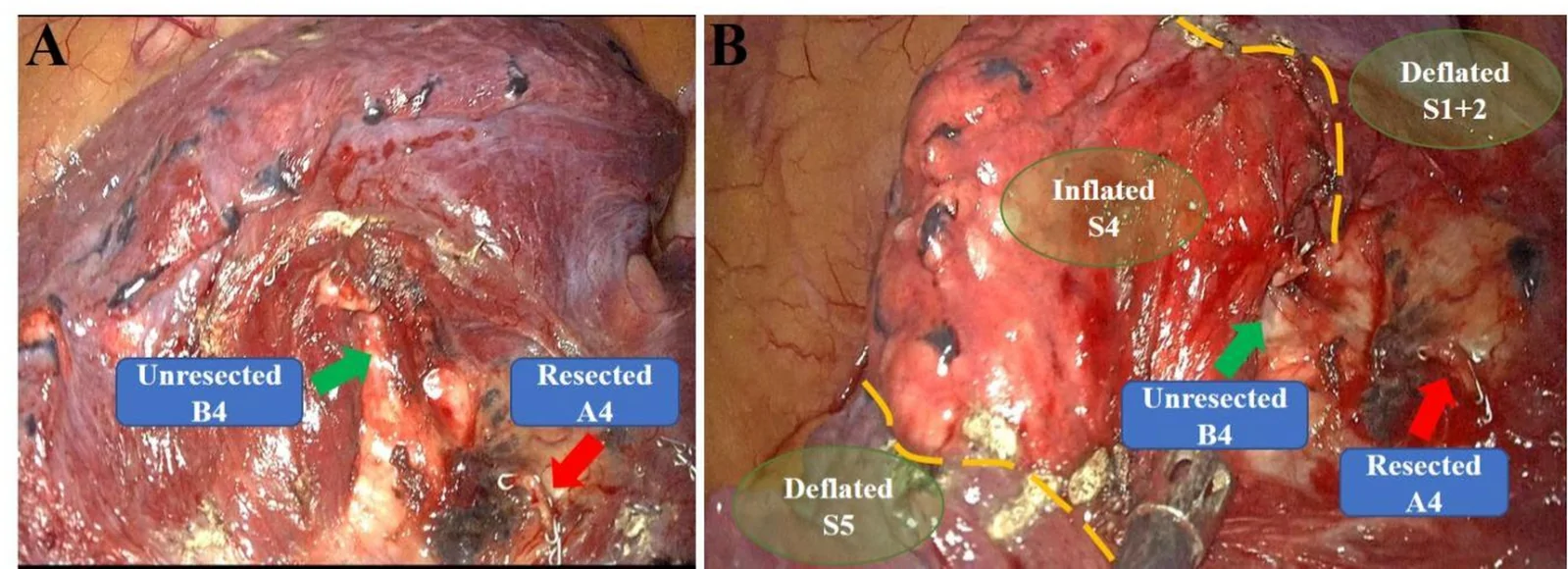
Procedure of the novel and modified inflation-deflation method. (**A**) The image before restoring bilateral lung ventilation. (**B**) The image after performing single lung ventilation again and waiting for about 10 min. B4, superior lingular bronchus; A4, superior lingular branch of left pulmonary artery; S4, superior lingular segment; S5, inferior lingular bronchus; S1 + 2, apicoposterior segment

During the operation, our team used a novel and simple inflation-deflation method to confirm the intersegmental border. Firstly, isolating and dividing the A4 (Fig. 3A). Secondly, the anesthetist restored bilateral lung ventilation and then performed the single lung ventilation again after the entire lung was inflated with pure oxygen. Finally, waiting patiently for about 10 min, a picture where S4 was still inflated and the rest of the lung collapsed was seen (Fig. 3B).

The operation was completed successfully and the patient was discharged from hospital on the fourth day after the operation without any complications.

## Discussion

The JCOG0802/WJOG4607L and CALGB140503 trials demonstrated that segmentectomy was non-inferior to lobectomy with respect to overall survival [13, 14]. JCOG0802/WJOG4607L showed a 3.5% difference in median FEV1 reduction at 1‑year follow‑up (*p* < 0.0001) between the two groups, which was below the 10% threshold for clinical significance [13]. CALGB140503 reported a 2‑percentage‑point higher predicted FEV1 in the sublobar resection group at 6 months after surgery [14]. A meta-analysis showed that compared with lobectomy, segmentectomy provided the better lung function preservation, with significantly milder declines in FEV1, FVC, FVC%, FEV1/FVC and DLCO [15]. Therefore, suitable candidates for pulmonary segmentectomy is importance. Because of the invisibility of intersegmental plane, the critical step of segmentectomy is how to confirm it precisely. Currently, there are alternative methods for thoracic surgeons, and they can be classified into two broad categories: Inflation-Deflation Line (IDL) method and Dyeing the Border (DB) method [3]. The modified Inflation-deflation method is a representative example of the former and is applied to the clinical practice as a standard technology. A novel and simple Inflation-deflation method mentioned in this article is the result of the improvement and simplification of modified Inflation-deflation method. Unlike the traditional method that involves dividing the targeted segmental bronchus, artery and vein, only the targeted segmental artery needs to be divided for this new method. On the one hand, we can use the time for waiting for intersegmental border to isolate other structures of segmental pedicle. On the other hand, the identification of intersegmental border can be useful to determine whether the isolated bronchus and vein are correct once again. Recently, the selective segmental inflation via bronchofiberoptic jet as another type IDL method is applied in clinical work [4, 5].

DB method is a technology combining fluorescent imaging with indocyanine green (ICG) to delineate the anatomical intersegmental line. Compared to IDL method, the relatively rapid appearance time of the intersegmental plane and stronger repeatability are merits of DB method. After intravenous systemic injection of ICG, the infrared thoracoscopy is safe and effective method to identify intersegmental plane without need for lung re-inflation [6–8]. And the intersegmental demarcation generated by the fluorescence imaging method was coincident with the modified Inflation-deflation method [9]. A different technique was reported in one experimental animal study and applied to identify the intersegmental line by using dye injection into the corresponding segmental artery [10]. Sekine Y and his colleagues thought the duration of intravascular ICG visualization is relatively short, and so proposed a novel method to determine the intersegmental plane by preoperative transbronchial ICG injection [11]. One thing to note here is that the injection rate of ICG should be controlled. If the injection is rapid, ICG will spread to the adjacent segments through the Kohn pores, and a wrong intersegmental plane will be showed [12]. Compared with DB method, this new method we proposed is simpler, easier to manage, easier to spread and does not need any additional auxiliary materials. However, in contrast to the DB method, the several inherent disadvantages of the new method could not be ignored. First, an obvious visualization of the intersegmental border may require an extra time, and the time is varied from case to case. Second, the performance of this method may be limited in patients with severe emphysema, an inherent drawback of the IDL method. Finally, for VATS, an inflated lung may obstruct the exposure of the operation field and increase the difficulty of surgery to a certain extent [3, 9, 11]. All methods have respective strengths and weaknesses, and the most suitable one should be chosen based on real clinical conditions.

We hypothesize that there are at least two mechanisms involved in the appearance of inflation-deflation line by new method; however, supporting references for this hypothesis remain absent in existing studies. On the one hand, because the targeted artery is divided, there is no blood for gas transfer with the target segment. Simultaneously, the pure oxygen in the preserved segments enters the circulation system due to gas transfer. On the other hand, the lung of surgical side is not ventilated because of single lung ventilation. The oxygen in the targeted segment is trapped inside, which caused the targeted segment remaining inflated. At the same time, due to the lack of gaseous exchange with the atmosphere, the oxygen in the preserved segments cannot be replenished, which resulting in a collapsed state of the preserved segments.

There are some limitations in this study. First, our study is just a case report and short of sufficient statistical data analysis to compare the difference between this new method and other methods. Besides, this case involves the superior lingular bronchus and whether other segments are suitable for this new method needs further clinical practice. What’s worse, the target segment of this case is supplied by single artery. If the anatomical variance of segment artery is relatively sophisticated, it is not clear whether this new method is feasible at the present stage.

## Conclusions

In conclusion, it is demonstrated in our study that this novel and simple Inflation-deflation method is effective to confirm the intersegmental demarcation, especially for segmentectomy with single targeted artery. And compared with other methods, this new method not only saves time and is easy to operate, but also identify targeted bronchus and vein again. However, this is our initial experience, and a further research should be performed to illustrate its clinical application value.

## Data Availability

All data generated or analyzed during this study are included in this published article.

## Declarations

### Data sharing and data accessibility

All data generated or analyzed during this study are included in this published article.
